# Visual EKF-SLAM from Heterogeneous Landmarks †

**DOI:** 10.3390/s16040489

**Published:** 2016-04-07

**Authors:** Jorge Othón Esparza-Jiménez, Michel Devy, José L. Gordillo

**Affiliations:** 1 Center for Robotics and Intelligent Systems, Tecnológico de Monterrey, Monterrey 64849, Mexico; jlgordillo@itesm.mx; 2 CNRS, LAAS, Université de Toulouse, 7 Avenue du Colonel Roche, Toulouse F-31400, France; michel.devy@laas.fr

**Keywords:** SLAM, EKF, computer vision, landmarks, points, lines

## Abstract

Many applications require the localization of a moving object, e.g., a robot, using sensory data acquired from embedded devices. Simultaneous localization and mapping from vision performs both the spatial and temporal fusion of these data on a map when a camera moves in an unknown environment. Such a SLAM process executes two interleaved functions: the front-end detects and tracks features from images, while the back-end interprets features as landmark observations and estimates both the landmarks and the robot positions with respect to a selected reference frame. This paper describes a complete visual SLAM solution, combining both point and line landmarks on a single map. The proposed method has an impact on both the back-end and the front-end. The contributions comprehend the use of heterogeneous landmark-based EKF-SLAM (the management of a map composed of both point and line landmarks); from this perspective, the comparison between landmark parametrizations and the evaluation of how the heterogeneity improves the accuracy on the camera localization, the development of a front-end active-search process for linear landmarks integrated into SLAM and the experimentation methodology.

## 1. Introduction

Simultaneous localization and mapping (SLAM) is an essential functionality required on a moving object for many applications where the localization or the motion estimation of this object must be determined from sensory data acquired by embedded sensors. The object is typically a robot or a vehicle, the position of which is required to deal with robust navigation in a cluttered environment. A SLAM module could also be required on smart tools (phones, glasses) to offer new services, e.g., augmented reality [1,2,3].

The robot or smart tool could be equipped with a global navigation satellite system (GNSS) receiver for outdoor applications to obtain directly a position with respect to the Earth reference frame [4]; at present, indoor localization with respect to a building reference frame could also be provided using ultra-wide band (UWB) [5], WiFi [6] or RF devices [7], on the condition that a hotspot or antenna network has been previously installed and calibrated. However, the direct localization is not always available (*i.e*., occlusions, bad propagation, multiple paths); so generally, they are combined using loose or tie fusion strategies, with motion estimates provided by an inertial measurement unit (IMU), integrating successive accelerometer and gyro data [8,9,10]. Nevertheless, even GPS-IMU fusion could fail or be too inaccurate. Depending on the context, *a priori* knowledge could be exploited; a map matching function can be sufficient, as in the GPS-based navigation systems available on commercial vehicles.

Considering mobile robots or the emerging autonomous vehicles, it is necessary to also make use of data acquired on the environment with embedded exteroceptive sensors, e.g., laser range finders [11], 3D sensors (ToF cameras [12], Kinect [13]) or vision with many possible modalities (mono, stereo, omni). Here, only visual SLAM is considered, due to the fact that it could be integrated both in low-cost unmanned ground and aerial vehicles and on smart tools equipped with cameras. Many visual SLAM methods have been proposed during the last decade [3,14].

**Figure 1:**
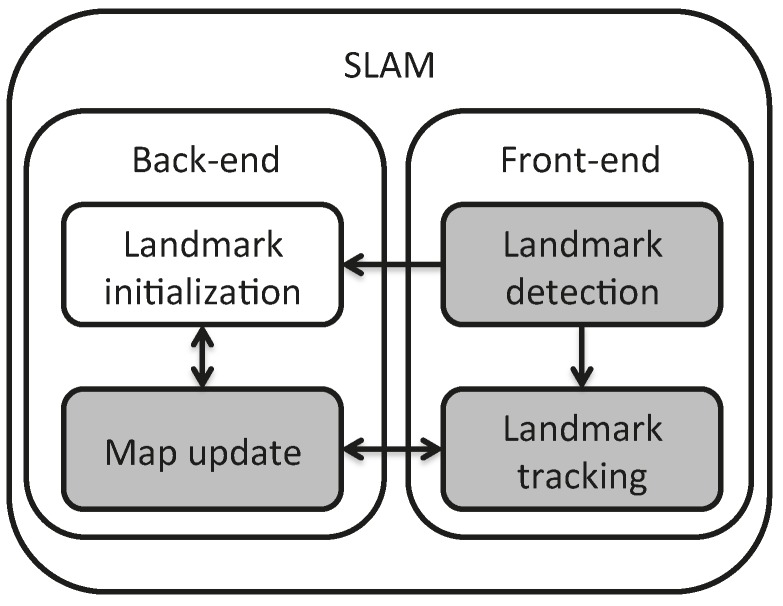
General block diagram of the SLAM solution. The blocks in gray represent the contributions of the present method.

A SLAM method combines two interleaved functionalities shown in Figure 1: the front-end detects and tracks features from images acquired from the moving robot, while the back-end, interpreting these feature and landmark observations, estimates both the landmark and robot positions with respect to the selected reference frame.

The back-end can be based either on estimation (Kalman [15], information [16], particle filters [17]) or optimization (bundle adjustment) frameworks [18]. The more classic landmarks are 3D points, detected as interest points (SIFT [19], SURF [20], FAST [21]), matched by using their descriptors (binary robust independent elementary features (BRIEF) [22]), or tracked by using Kanade-Lucas-Tomasi (KLT) feature tracker [23], or an active-search strategy [24]. Generally, the set of 3D points extracted from an image does not give any semantic information, unlike 3D lines, which correspond generally to sharp 3D edges in the environment. This is the reason why segment-based SLAM from either an estimation [25] or optimization [26] back-end, has been proposed. The main challenge of these methods concerns the front-end, *i.e.*, the robustness of the line detection and tracking in successive images.

The initialization of such landmarks with their minimal Euclidean parameters requires more than one observation. One way to solve this problem was the delayed initialization [3,27], in which a landmark was added to the map only when it was known in the Euclidean space. This does not allow use of landmarks that are very far from the robot. An alternative solution is to add them to the map, as soon as they are observed (*i.e.*, undelayed initialization), and it has been proposed for point [28,29] or line [25] landmarks. The pros and cons of several representations for 3D points and 3D lines have been analyzed in [30].

This article is devoted to the analysis of a visual SLAM solution using a heterogeneous map, as a more complete approach where points and lines are both included from features extracted in images acquired by a camera moving in the environment, and with undelayed initialization. Therefore, the contributions of the proposed method comprehend the use of heterogeneous landmark-based SLAM (the management of a map composed of heterogeneous landmarks), and from this perspective, the comparison between landmark parametrizations, the development of a front-end active-search process for linear landmarks integrated into SLAM (the processing of linear landmarks by the inclusion of detection and tracking algorithms taken from the state of the art) and the experimentation methodology. These contributions correspond to the gray blocks in Figure 1. Only the first two contributions were covered in [31], which dealt with the information fusion back-end for the map computation. The present article extends this concept to its application on real images, by developing the proposed front-end. Even if optimization-based methods make it possible to avoid the possible divergence of methods based on estimation due to linearization of the observation model, the fusion is performed from an extended Kalman filter, as a very light approach that can be integrated into a dedicated architecture to be used on small aerial vehicles.

Section 2 covers the techniques and parametrizations for the initialization and update of point and line landmarks on the map. Section 3 describes the detection and tracking methods selected and developed for the front-end. Section 4 focuses on experiments, including a simulation part that compares landmark representations, a real image part that recalls the integration of segments with the already existing point-based front-end and an integral experiment. The results of the experiments are presented and discussed in Section 5. Finally, Section 6 offers some conclusions.

## 2. SLAM Back-End

The SLAM back-end deals with the initialization of observed features as landmarks on the map and the estimation or update of both the landmarks and the robot positions with respect to a selected reference frame. This section deepens the description of each landmark parametrization, the initialization and update algorithms and it derives from the theoretical study presented in [31].

The undelayed landmark initialization (ULI) is presented in [30] for different point and line parametrizations. The main idea is to model the uncertainty derived from unmeasured degrees of freedom by a Gaussian prior that handles unbounded limits, in a manner that can be handled by EKF.

The implications of uncertainty are different for point and line landmarks. For points, depth is initially unknown, and uncertainty is present along the visual ray until infinity. In the case of infinite straight lines, uncertainty is present in two degrees of freedom, corresponding to a depth that should be covered up to infinity and all possible orientations.

### 2.1. 3D Point Parametrizations

In this section, a Euclidean point is described as a reference, for subsequent covering of the ones used for initialization purposes (*i.e.*, homogeneous point and anchored homogeneous point). Each description includes camera projection and back-projection.

**Figure 2:**
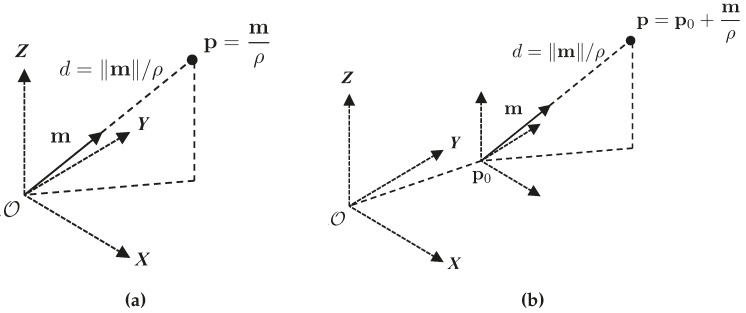
Point parametrizations used for initialization. (**a**) Homogeneous point parametrization; (**b**) anchored homogeneous point parametrization.

Point parametrizations used for initialization are shown in Figure 2.

#### 2.1.1. Euclidean Point

A Euclidean point is parametrized by its Cartesian coordinates.

$$L_{EP}=p={\left[x\;y\;z\right]}^{T}\in R^{3}$$

The projection to the camera frame is performed as follows:

(1) $$\underline{u}=KR^{T}\left(p-T\right)\in P^{2}$$

where,

$$K=\left[\begin{array}{ccc} \alpha_{u} & 0 & u_{0}\\ 0 & \alpha_{v} & v_{0}\\ 0 & 0 & 1 \end{array}\right]$$

is the intrinsic parameter matrix and R and T are the rotation matrix and translation vector that define the camera C. Homogeneous coordinates are represented by underlined vectors, like $\underline{u}$.

#### 2.1.2. Homogeneous Point

Homogeneous points are four-vector composed of the 3D vector **m** and scalar *ρ*, as introduced in [32].

$$L_{HP}=\underline{p}=\left[\begin{array}{c} m\\ \rho \end{array}\right]={\left[m_{x}\;m_{y}\;m_{z}\;\rho \right]}^{T}\in P^{3}\subset R^{4}$$

The vector **m** gives the direction from the origin O to the point **p**, while *ρ* serves as a scale factor for providing the magnitude for each coordinate of the point.

The conversion from homogeneous to Euclidean coordinates is given by the following equation:

(2) $$p=\frac{m}{\rho }$$

Depending on the characteristics of the parameters **m** and *ρ*, there are three different canonical representations for a homogeneous point. The original Euclidean point refers to the case when *ρ* = 1, inverse-depth has $m_{z}=1$ and in inverse-distance ∥m∥=1.

In the camera frame, **m** is the director vector of the optical ray, and *ρ* has a linear dependence on the inverse of the distance *d* defined from the optical center to the point:

$$\rho =\frac{∥m∥}{d}$$

The unbounded distance of a point along the optical ray from zero to infinity can then be expressed in the bounded interval in parameter space $\rho \in \left(0,∥m∥/d_{min}\right]$.

The frame transformation of a homogeneous point is performed according to the next equation:

(3) $$\underline{p}=H{\underline{p}}^{C}=\left[\begin{array}{cc} R & T\\ 0 & 1 \end{array}\right]{\underline{p}}^{C}$$

where super-index C indicates the frame to which the point is referred and matrix **H** specifies the frame to which the point is transformed.

The projection of a homogeneous point into the image frame is performed with the following equation:

(4) $$\underline{u}=KR^{T}\left(m-T\rho \right)\in P^{2}$$

By expressing a homogeneous point in the camera frame, the projected image point is $\underline{u}={\mathrm{Km}}^{C}$, where super-index C indicates the frame to which the point is referred. In this case, $\rho^{C}$ is not measurable. Back-projection is then:

$$m^{C}=K^{-1}\underline{u}$$

The complete homogeneous point parametrization is the following:

(5) $$\begin{array}{c} L_{HP}=\underline{p}=\left[\begin{array}{c} m\\ \rho \end{array}\right]=H\left[\begin{array}{c} K^{-1}\underline{u}\\ \rho^{C} \end{array}\right]=\left[\begin{array}{c} RK^{-1}\underline{u}+T\rho^{C}\\ \rho^{C} \end{array}\right] \end{array}$$

where $\rho^{C}$ must be given as a prior and represents the inverse-distance from the origin of the coordinates, that is the scalar value that makes ∥m∥=1.

#### 2.1.3. Anchored Homogeneous Point

Linearity is supposed to be improved by the addition of an anchor that serves as a reference to the optical center at the initialization time of the landmark. The landmark is then composed of seven elements that include Cartesian coordinates of the anchor, the point with respect to the anchor and an inverse-distance scalar.

$$L_{AHP}=\left[\begin{array}{c} p_{0}\\ m\\ \rho \end{array}\right]={\left[x_{0}\;y_{0}\;z_{0}\;m_{x}\;m_{y}\;m_{z}\;\rho \right]}^{T}\in R^{7}$$

The conversion from the anchored homogeneous point to Euclidean coordinates can be obtained by the following equation:

(6) $$p=p_{0}+\frac{m}{\rho }$$

The projection and frame transformation process is given below:

(7) $$\underline{u}=KR^{T}\left(m-\left(T-p_{0}\right)\rho \right)\in P^{2}$$

The anchor is chosen to be the position of the optical center at the initialization time, given by T. That way, the term multiplying the unmeasured degree of freedom *ρ* (*i.e.*, $\left(T-p_{0}\right)\rho$) is small after initialization. This helps to decouple the uncertainty of the most uncertain parameter *ρ*. The complete anchored homogeneous point parametrization for back projection and transformation is the following:

(8) $$L_{AHP}=\left[\begin{array}{c} p_{0}\\ m\\ \rho \end{array}\right]=\left[\begin{array}{c} T\\ RK^{-1}\underline{u}\\ \rho^{C} \end{array}\right]$$

where $\rho^{C}$ must be given as the prior.

### 2.2. 3D Line Parametrizations

The line parametrization includes the projection to the image frame and back-projection to 3D.

**Figure 3:**
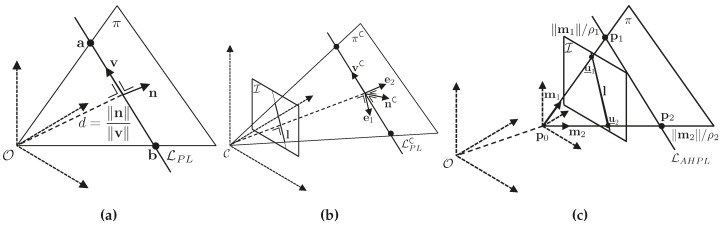
Line parametrizations used for initialization. (**a**) Plücker line geometrical representation; (**b**) Plücker line back-projection; (**c**) anchored homogeneous-points line parametrization.

The Plücker line and anchored homogeneous point line are shown in Figure 3.

#### 2.2.1. Plücker Line

Plücker coordinates are conformed by a six-vector that represents a line in $P^{3}$ defined by two points $\underline{a}={\left[a\;a\right]}^{T}$ and $\underline{b}={\left[b\;b\right]}^{T}$:

$$L_{PL}=\left[\begin{array}{c} n\\ v \end{array}\right]={\left[n_{x}\;n_{y}\;n_{z}\;v_{x}\;v_{y}\;v_{z}\right]}^{T}\in P^{5}\subset R^{6},$$

where n=a×b, v=ab−ba, $n,v\in R^{3}$, with the Plücker constraint: $n^{T}v=0$.

In terms of geometry, **n** is the vector normal to the plane *π* containing the line and the origin and **v** is the director vector from $\underline{a}$ to $\underline{b}$. The Euclidean orthogonal distance from the line to the origin is given by ∥n∥/∥v∥. Hence, ∥v∥ is the inverse-distance, analogous to *ρ* of homogeneous points. Plücker line geometrical representation is shown in Figure 3a.

Expressions for transformation and inverse-transformation of Plücker coordinates from and to the camera frame are as shown next:

(9) $$\begin{array}{ccc} L_{PL} & = & H\cdot L_{PL}^{C}=\left[\begin{array}{cc} R & {\left[T\right]}_{\times }R\\ 0 & R \end{array}\right]\cdot \left[\begin{array}{c} n^{C}\\ v^{C} \end{array}\right] \end{array}$$

(10) $$\begin{array}{ccc} L_{PL}^{C} & = & H^{-1}\cdot L_{PL}=\left[\begin{array}{cc} R^{T} & -R^{T}{\left[T\right]}_{\times }\\ 0 & R^{T} \end{array}\right]\cdot \left[\begin{array}{c} n\\ v \end{array}\right] \end{array}$$

The transformation and projection process in terms of R, T, **n** and **v** is as follows:

(11) $$l=K\cdot R^{T}\cdot \left(n-T\times v\right)$$

where the intrinsic projection Plücker matrix K is defined as:

$$K=\left[\begin{array}{ccc} \alpha_{v} & 0 & 0\\ 0 & \alpha_{u} & 0\\ -\alpha_{v}u_{0} & \alpha_{u}v_{0} & \alpha_{u}\alpha_{v} \end{array}\right]$$

When Plücker coordinates are expressed in the camera frame, projection is obtained by:

(12) $$l=K\cdot n^{C}$$

The range and orientation of the line are included in $v^{C}$ and are not measurable.

For Plücker line back projection, vectors $n^{C}$ and $v^{C}$ are obtained with these expressions:

$$\begin{array}{cc} n^{C} & =K^{-1}\cdot l\\ v^{C} & =\beta_{1}\cdot e_{1}+\beta_{2}\cdot e_{2} \end{array}$$

where $\beta_{1},\beta_{2}\in R$ and $\left\{e_{1},e_{2},n^{C}\right\}$ are mutually orthogonal.

Defining $\beta =\left(\beta_{1},\beta_{2}\right)\in R^{2}$, vector $v^{C}$ can also be expressed as:

$$v^{C}=E\cdot \beta ,$$

where $v^{C}\in \pi^{C}$ for any value of *β*. Plücker line back projection is shown in Figure 3b.

The complete Plücker line parametrization for back projection and transformation is given in the following equation:

(13) $$L_{PL}=H\left[\begin{array}{c} n^{C}\\ v^{C} \end{array}\right]=H\left[\begin{array}{c} K^{-1}l\\ E\beta \end{array}\right]=\left[\begin{array}{c} RK^{-1}l+T\times RE\beta \\ RE\beta \end{array}\right]$$

where *β* must be provided as a prior.

#### 2.2.2. Anchored Homogeneous Points Line

A line can also be represented by the end points defining it. With the application of the anchored homogeneous point parametrization, shown in Figure 3c, an anchored homogeneous-points line is an eleven-vector defined as follows:

$$L_{AHPL}={\left[p_{0}\;m_{1}\;\rho_{1}\;m_{2}\;\rho_{2}\right]}^{T}\in R^{11}$$

For each point, the transformation and projection of a pinhole camera is as previously stated in Equation (4).

A homogeneous 2D line is obtained by the cross product of two points lying on it, $l={\underline{u}}_{1}\times {\underline{u}}_{2}$, giving:

(14) $$l=KR^{T}\left(\left(m_{1}\times m_{2}\right)-\left(T-p_{0}\right)\times \left(\rho_{1}m_{2}-\rho_{2}m_{1}\right)\right)$$

In comparison to the result obtained for Plücker coordinates, the product $m_{1}\times m_{2}$ is a vector orthogonal to the plane *π*, analogous to the Plücker sub-vector **n**. Furthermore, the term $\left(\rho_{1}m_{2}-\rho_{2}m_{1}\right)$ is a vector that gives the direction between the points of the line, therefore related to Plücker sub-vector **v**.

The complete anchored homogeneous point line parametrization for back projection and transformation is the following:

(15) $$L_{AHPL}=\left[\begin{array}{c} p_{0}\\ m_{1}\\ \rho_{1}\\ m_{2}\\ \rho_{2} \end{array}\right]=\left[\begin{array}{c} T\\ RK^{-1}{\underline{u}}_{1}\\ \rho_{1}^{C}\\ RK^{-1}{\underline{u}}_{2}\\ \rho_{2}^{C} \end{array}\right]$$

where $\rho_{1}^{C}$ and $\rho_{2}^{C}$ must be given as priors.

### 2.3. Landmark Initialization

The process of the initialization of a landmark consists of the detection of a feature in the image, retro-projection to 3D and inclusion into the map. There are three important concepts that are involved in the landmark initialization: the 3D landmark **x** itself, the 2D measurement **z** of the landmark in the image and the unmeasured degree of freedom *π*. All of these are modeled as Gaussian variables, whose notation is $N\left\{\mu ,\sigma^{2}\right\}$. Thus, the cited concepts are expressed as $x∼N\left\{\bar{x},P\right\}$, $z∼N\left\{\bar{z},R\right\}$ and $\pi ∼N\left\{\bar{\pi },\Pi \right\}$, respectively. The 3D landmarks **x** considered for this study are points and lines, already described in Section 2.1 and Section 2.2. The parametrizations for landmark 2D measurements **z** and unmeasured degrees of freedom *π*, as well as the description of the initialization algorithm are covered in the following sections.

#### 2.3.1. Landmark 2D Measurements in the Image

Points are represented as a two-vector containing Cartesian coordinates in pixel space, leading to the following:

$$u=\left[\begin{array}{c} u\\ v \end{array}\right]∼N\left\{\bar{u},U\right\},$$

where **U** is the covariance matrix of the position of the point.

In homogeneous coordinates,

$$\underline{u}=\left[\begin{array}{c} u\\ 1 \end{array}\right]∼N\left\{\underline{\bar{u}},\underline{U}\right\}=N\left\{\left[\begin{array}{c} \bar{u}\\ 1 \end{array}\right],\left[\begin{array}{cc} U & 0\\ 0 & 0 \end{array}\right]\right\}$$

Lines can be expressed by a four-vector that represents the coordinates of their end-points, also with a Gaussian probability density function.

$$s=\left[\begin{array}{c} u_{1}\\ u_{2} \end{array}\right]∼N\left\{\bar{s},S\right\}=N\left\{\left[\begin{array}{c} {\bar{u}}_{1}\\ {\bar{u}}_{2} \end{array}\right],\left[\begin{array}{cc} U & 0\\ 0 & U \end{array}\right]\right\}$$

The probability density function for infinite lines like Plücker, $N\left\{\bar{l},L\right\}$, is composed of the homogeneous line representation and the covariance matrix defined as follows:

$$\begin{array}{cc} \bar{l} & ={\bar{\underline{u}}}_{1}\times {\bar{\underline{u}}}_{2},\mathrm{and}\\ L & ={\left[{\bar{\underline{u}}}_{1}\right]}_{\times }\underline{U}{\left[{\bar{\underline{u}}}_{1}\right]}_{\times }^{T}+{\left[{\bar{\underline{u}}}_{2}\right]}_{\times }\underline{U}{\left[{\bar{\underline{u}}}_{2}\right]}_{\times }^{T} \end{array}$$

#### 2.3.2. Unmeasured Degrees of Freedom

The uncertainty in 3D points and lines coming from projection is represented by inverse-distance variables $\rho^{C}$ and $\beta^{C}$, which are modeled as Gaussian variables. The origin of each of these priors must be inside the 2σ of their probability density functions.

For points and end-point-based lines, the minimum distance must match the upper 2σ boundary, hence:

$$\begin{array}{cc} \rho & -n\sigma_{\rho }=0,\;0\leq n<2\\ \rho & +2\sigma_{\rho }=1/d_{\min} \end{array}$$

Then, n=1 leads to,

$$\bar{\rho }=1/3d_{\min}\;and\;\sigma_{\rho }=1/3d_{\min}$$

The probability density function of a point based line is defined as $t^{C}∼N\left\{\bar{t},T\right\}$, with:

$$\bar{t}=\left[\begin{array}{c} \bar{\rho }\\ \bar{\rho } \end{array}\right],T=\left[\begin{array}{cc} \sigma_{\rho }^{2} & 0\\ 0 & \sigma_{\rho }^{2} \end{array}\right]$$

The Plücker lines prior $\beta^{C}∼N\left\{\bar{\beta },B\right\}$ take the following values:

$$\bar{\beta }=\left[\begin{array}{c} 1/3d_{\min}\\ 0 \end{array}\right],\;\text{and}\;B=\left[\begin{array}{cc} {\left(1/3d_{\min}\right)}^{2} & 0\\ 0 & {\left(1/2d_{\min}\right)}^{2} \end{array}\right]$$

This initializes lines at the front of the camera.

#### 2.3.3. Undelayed Landmark Initialization Algorithm

The ULI algorithm was presented in [30] for the construction of landmark-based stochastic maps, including a single type of landmark L. The approach presented in this paper includes heterogeneous parametrizations of landmarks $L_{*}$ on the same map, where $L_{*}$ can be a point or line. The resulting algorithm is composed of the following steps:

1. Identify mapped magnitudes $x∼N\left\{\bar{x},P\right\}$.
2. Identify measurements $z∼N\left\{\bar{z},R\right\}$, where z is either a point or a line (*i.e.*, u or s, respectively).
3. Define Gaussian prior $\pi ∼N\left\{\bar{\pi },\Pi \right\}$ for the unmeasured degree of freedom. *π* can either be $\rho^{C}$, $t^{C}$ or $\beta^{C}$.
4. Back-project the Gaussian measurement, and get the landmark mean and Jacobians.
  $$\begin{array}{c} \bar{L_{*}}=g\left(\bar{C},\bar{z},\bar{\pi }\right)\\ G_{C}={\left.\frac{dg}{dC}\right|}_{\bar{C},\bar{z},\bar{\pi }},G_{z}={\left.\frac{dg}{dz}\right|}_{\bar{C},\bar{z},\bar{\pi }},G_{\pi }={\left.\frac{dg}{d\pi }\right|}_{\bar{C},\bar{z},\bar{\pi }}, \end{array}$$
  where g() is the back projection and transformation function for the corresponding landmark. C=(T,Q) is the camera frame expressed in terms of its position T and orientation Q in quaternion nomenclature.
5. Compute landmarks’ co- and cross-variances.
  $$\begin{array}{c} P_{L}*L_{*}=G_{C}P_{\mathrm{CC}}G_{C}^{T}+G_{z}{\mathrm{RG}}_{z}^{T}+G_{\pi }\Pi G_{\pi }^{T}\\ P_{L_{*}x}=G_{C}P_{Cx}=G_{C}\left[P_{\mathrm{CC}}\;P_{\mathrm{CM}}\right] \end{array}$$
6. Augment the SLAM map.
  $$\begin{array}{c} \bar{x}\leftarrow \left[\begin{array}{c} \bar{x}\\ L_{*} \end{array}\right]\\ P\leftarrow \left[\begin{array}{cc} P & P_{L_{*}x}^{T}\\ P_{L_{*}x} & P_{L_{*}L_{*}} \end{array}\right] \end{array}$$

### 2.4. Landmark Update

The purpose of the landmark update process is to recalculate the parameters of the elements on the map, (*i.e.*, the robot and landmark poses), given the observation of the already mapped landmarks in the current frame. This process starts by projecting all of the observable landmarks to the image plane and selecting those with higher uncertainty for correction. For points, the observation function *h*() applies a homogeneous to Euclidean transformation *h2e*() once having performed the projection process previously explained, as follows:

$$z=h2e\left(\underline{u}\right)=\left[\begin{array}{c} u_{1}/u_{3}\\ u_{2}/u_{3} \end{array}\right]\in R^{2}$$

Innovation mean **y** and covariance **Y** is then obtained as shown next:

$$\begin{array}{cc} y & =z-h\left(\bar{x}\right)\\ Y & =R+H\cdot P\cdot H^{T}, \end{array}$$

where R=U is the measurement noise covariance and Jacobian $H\;=\;{\left.\frac{\partial h}{\partial x}\right|}_{\bar{x}}$.

**Figure 4:**
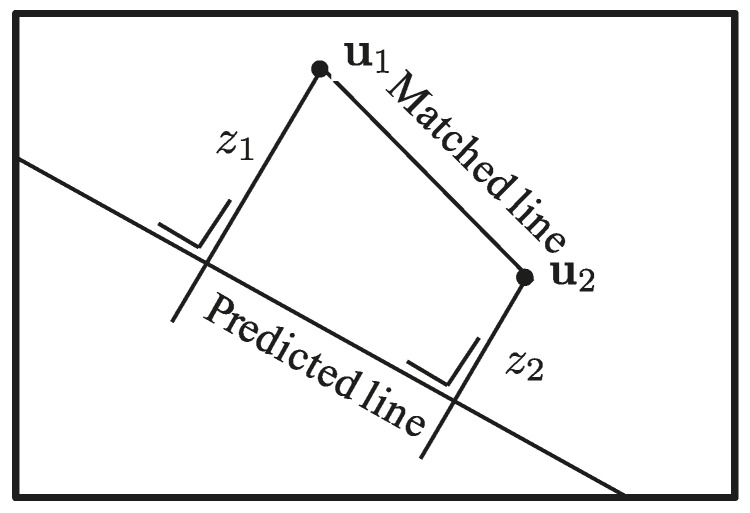
Measurement of the orthogonal distances from the detected end-points to the expected (or predicted) line.

For lines, the innovation function computes the orthogonal distances from the detected end-points $u_{i}$ to a line **l**, as shown in Figure 4, leading to the following:

(16) $$z=\left[\begin{array}{c} l^{T}\cdot {\underline{u}}_{1}/\sqrt{l_{1}^{2}+l_{2}^{2}}\\ l^{T}\cdot {\underline{u}}_{2}/\sqrt{l_{1}^{2}+l_{2}^{2}} \end{array}\right]\in R^{2}$$

Since the EKF innovation is the difference between the actual measurement and the expectation and **z** is the orthogonal distance previously described, the line innovation function is:

y=0−z,

as the desired orthogonal distance from the predicted line to the matched end-points is zero.

A landmark is found consistent if the squared Mahalanobis distance *MD*2 of innovation is smaller than a threshold *MD*2*th*.

$$MD2=y^{T}\cdot Y^{-1}\cdot y<MD2th$$

As that is true, the landmark is updated:

$$\begin{array}{cc} \text{Kalman gain}: & K=P\cdot H\cdot Y^{-1}\\ \text{State update}: & \bar{x}\leftarrow \bar{x}+K\cdot y\\ \text{Covariance update}: & P\leftarrow P-K\cdot H\cdot P \end{array}$$

Point and line parametrizations are modeled as Gaussian variables in [25,29,30], validating the use of Mahalanobis distance as compared to a chi-squared distribution. Kalman gain is assumed to be optimal. Since this process is intended to be developed as a light approach that could be integrated into a dedicated architecture on small vehicles, the selected covariance update formula is used instead of the Joseph form, which has such a high complexity that it may compromise performance. Successful results of this formulation are presented in [30].

## 3. SLAM Front-End

To obtain the geometrical representation of landmarks given by the SLAM back-end, it is necessary to process the information coming from the sensors embedded in the moving agent (*i.e.*, cameras mounted on the mobile robot). The front-end deals with the detection of new landmarks and the matching of already existent ones in subsequent images.

This section covers the image processing algorithms used for detecting and matching points and lines. Points have been widely studied and implemented as SLAM landmarks [3,24,33,34]. In the case of lines, a different front-end strategy was integrated for the detection and tracking of line segments.

### 3.1. Point Landmarks

A point landmark is modeled as an appearance descriptor composed of a patch of pixels around the point in the image. Once detected, the patch is used for the matching of the feature on incoming images.

#### 3.1.1. Point Detection

**Figure 5:**
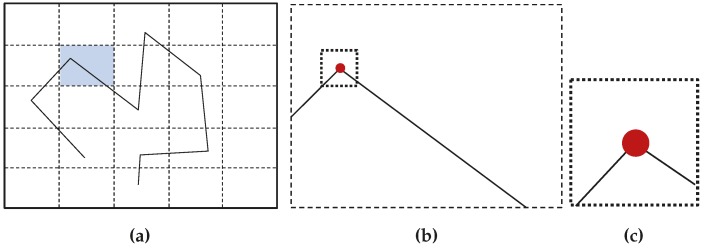
Point detection process. (**a**) Image divided into regions; one is randomly selected; (**b**) selected region, processed to find a corner point; (**c**) appearance of the point.

An active-search approach [24,34] can ensure that the point landmarks are equally distributed in the image by dividing it into a number of equal regions, in which it is expected to have a landmark (Figure 5a). At each iteration, an empty region is randomly selected, and a corner point is chosen to be the strongest Harris point [35] (Figure 5b). This point is used for the landmark initialization, and its appearance and the current position and orientation of the camera $C_{0}=\left(T_{0},R_{0}\right)$ are saved. The appearance of the point is given by the patch of pixels surrounding it, as seen in Figure 5c.

#### 3.1.2. Point Matching

**Figure 6:**
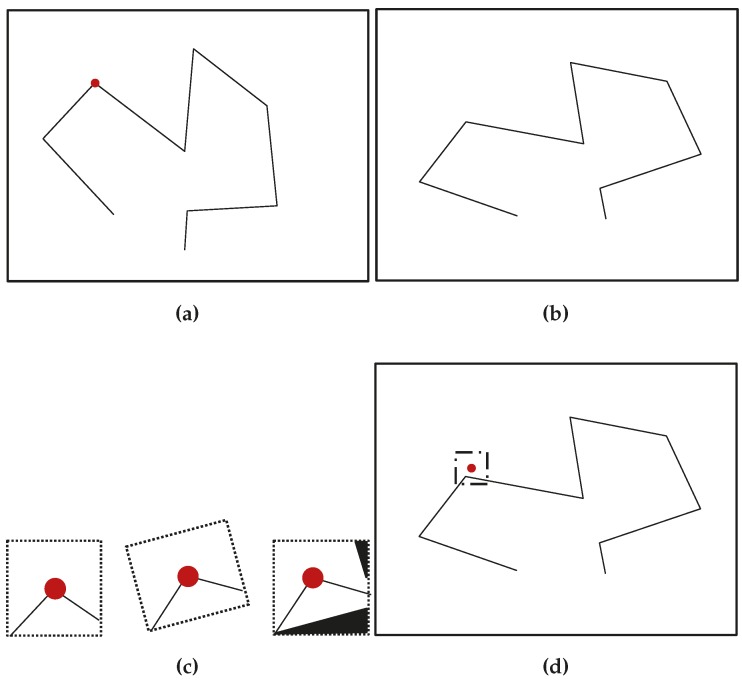
Point matching process. (**a**) Frame captured at camera pose $C_{0}$, where point x was initialized; (**b**) frame captured at camera pose $C_{i}$, where point **x** is intended to be matched; (**c**) patch warping process, showing the original, the transformed and the cropped patches; (**d**) projection of landmark point **x** into image point **u**_*i*_ on the image at camera pose $C_{i}$. The search area is indicated by the rectangle surrounding the point.

When there are point landmarks already mapped, the matching process searches for a point landmark **x** in the frame captured at camera pose $C_{i}$. This point has been initialized in the frame captured with camera pose $C_{0}$ (Figure 6a,b).

The saved appearance patch of the landmark is warped by applying a homography transformation. This transformation takes into account the rotation and translation in the camera position and orientation, with respect to its pose when the landmark was detected. The transformed coordinates of pixel *j* of the patch at camera pose *i* (*i.e.*, $q_{i}^{j}$) is computed as follows:

(17) $$q_{i}^{j}=Hq_{0}^{j}$$

where:

$$H=K\left[\begin{array}{cc} R & T\\ 0 & 1 \end{array}\right]K^{-1}$$

and $q_{0}^{j}$ are the coordinates of pixel *j* of the original patch, as R and T are the rotation matrix and translation vector between camera pose $C_{0}$ and camera pose $C_{i}$. Once the patch is warped, it is cropped to be squared in order to maintain the same dimensions of the original. This warping process is shown in Figure 6c.

The matching process performs the projection of point landmark **x** into the image at camera pose $C_{i}$ to get a point expected position $u_{i}$, given the current pose *i* of the camera:

$$u_{i}=h\left(C_{i},x\right)$$

The 2D covariance matrix **U** of this point is obtained from the 3D covariance matrix $P_{LL}$ corresponding to landmark L, as follows:

$$U=\left[\begin{array}{ccc} U_{\mathrm{RF}} & U_{\mathrm{SF}} & U_{L} \end{array}\right]P_{\mathrm{LL}}{\left[\begin{array}{ccc} U_{\mathrm{RF}} & U_{\mathrm{SF}} & U_{L} \end{array}\right]}^{T}$$

where $U_{\mathrm{RF}}$, $U_{\mathrm{SF}}$ and $U_{L}$ are the Jacobians of the projection **u** with respect to the robot frame, the sensor frame and the landmark, respectively.

Then, the zero mean normalized cross-correlation (ZNCC) test [36] is applied to the warped patch and a region of pixels surrounding the expected point in the image (Figure 6d). The rectangular search region is based on the projection mean **u** and the covariance ellipse **U**. The mean is the center of the search box, and the square roots of the diagonals of the covariance are the standard deviations, $\sigma_{u}$ and $\sigma_{v}$. The search region goes ±3σ at each side of the center. If the ZNCC score is over a threshold, the point is said to be matched.

### 3.2. Line landmarks

Many methods have been proposed to extract lines in the image processing community, generally starting from the detection of intensity discontinuities (gradient, Laplacian, Canny filter). The first method, introduced in the 1980s, was the chaining method [37], based on the polygonal approximation of extracted contours. This method is efficient, but the result is too dependent on its parametrizations (gradient threshold, contour thinning). This is why the Hough transform became so popular [38]; a recent variant, the kernel-based Hough transform [39], has been implemented and evaluated for line detection. However, this approach, when working with infinite lines rather than segments, makes its performance less than optimal for the intended purposes. In [40], the Dseg algorithm is proposed, close to the chaining method, but using an iterative filtering approach to integrate a contour point in the processed segment. The Dseg algorithm was compared to the chaining method, with the Hough transform and with the line segment detector (LSD) detector [41]; it was found that it allows extraction of a greater number of segments of various lengths.

This section covers the description of a front-end line segment active-search process, developed for segment-based SLAM. The selected techniques for working with segments are the LSD for the detection and moving edges (ME) [42] for matching.

An active-search approach was developed and implemented in order to handle the line segment landmarks, similar to the one previously described for points.

#### 3.2.1. Segment Detection

**Figure 7:**
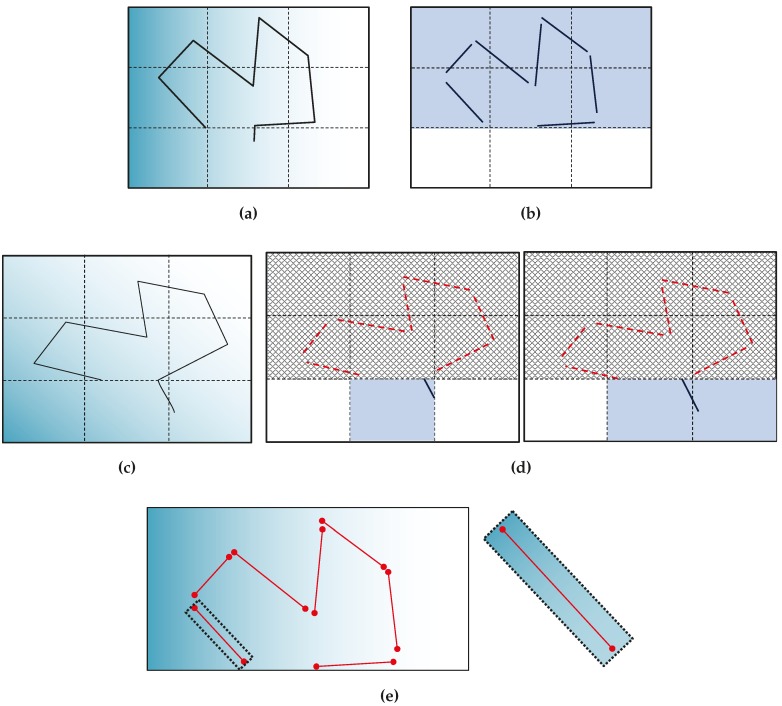
Line detection process. (**a**) Initial image divided in a 3 × 3 grid of cells. (**b**) Line segments detected. Only the ones that are longer than a threshold are selected. The cells that contain them are marked as “occupied”. (**c**) Later image of the sequence. The map already contains line landmarks. (**d**) Existing landmarks are projected into the image, and the occupation grid is updated. Occupied grids are marked with a textured pattern. The first image also shows the randomly-selected empty cell and the segment detected on it. The second image shows the result of extending the detected segment. Cells occupied by this segment are colored in blue. (**e**) Appearance of the detected lines. Each line is parametrized by its end points, the pixels surrounding the line are used for matching.

The process starts by building a grid that divides the image into rectangular cells. A 3 × 3 grid was chosen, as shown in Figure 7a. There are two different ways of detecting lines: the one applied in the first frame of the sequence, and the one used in all other frames. In the first case, the segment detection algorithm is run for the whole image, and the longest segments found are selected. The cells containing a whole or partial segment are marked as “occupied”. This is shown in Figure 7b. The detection process, applied in a subsequent frame (Figure 7c), departs from the assumption that there are line landmarks already on the map. Once the EKF back-end computes the 3D position of the robot and the landmarks seen so far, landmarks are projected to the image. The projection of line landmark **x** into the image at camera pose $C_{i}$ to get a line segment’s expected position $s_{i}$, given the current pose *i* of the camera, is performed as follows:

(18) $$s_{i}=h\left(C_{i},x\right)$$

Each projection is taken into account for updating the grid. The occupied cells are not considered, and one empty cell is chosen randomly. The image patch delimited by this cell is used to run the segment detector and to find the longest segment on it for initializing a new landmark. The line detected is extended to the other cells, and they are marked as “occupied” when this is the case. This process is shown in Figure 7d.

The patch of the cells where lines were detected in the present frame is saved for the line matching process, as well as the current position and orientation of the camera $C_{0}=\left(T_{0},R_{0}\right)$. Each line is defined by its end points, and only the pixels surrounding it are used for matching, as can be seen in Figure 7e.

#### 3.2.2. Segment Matching

**Figure 8:**
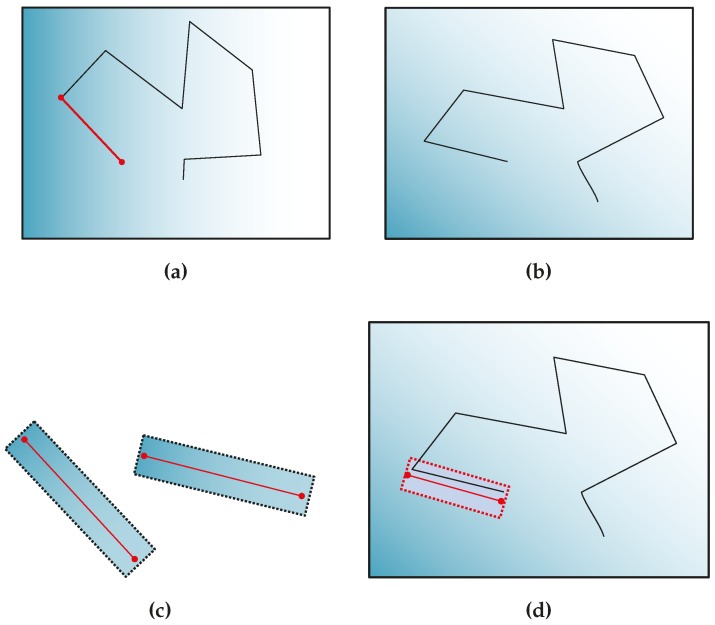
Line matching process. (**a**) Frame captured at camera pose $C_{0}$, where line **x** was initialized; (**b**) frame captured at camera pose $C_{i}$, where line **x** is intended to be matched; (**c**) patch warping process, showing the original and the transformed patches; (**d**) projection of landmark line **x** into image segment **s**_*i*_ on the image at camera pose $C_{i}$. The search area is indicated by the rectangle surrounding the segment.

When there are line landmarks already mapped, the matching process searches for a line landmark **x** in the frame captured at camera pose $C_{i}$. This line has been initialized in the frame captured with camera pose $C_{0}$ (Figure 8a,b).

The saved appearance patch of the landmark is warped by applying a homography transformation. This transformation takes into account the rotation and translation in the camera position and orientation, with respect to its pose when the landmark was detected. The transformed coordinates of pixel *j* of the patch at camera pose *i* (*i.e.*, $q_{i}^{j}$) are computed applying Equation (17). This warping process is shown in Figure 8c.

The matching process performs the projection of the line landmark from Equation (18) to estimate its position of line segment $s_{i}$ in the image. For updating the current position of each line landmark, their estimated position in the image is used for initializing the matching algorithm, and a search for a match is performed in the region surrounding the line estimation (Figure 8d). The errors between the estimated position and the position of the match found are used by the back-end to update the state and uncertainties of the landmarks and robot poses on the map.

To perform the tracking of the points that make up part of line segments, the moving edges algorithm is implemented as discussed in [43].

**Figure 9:**
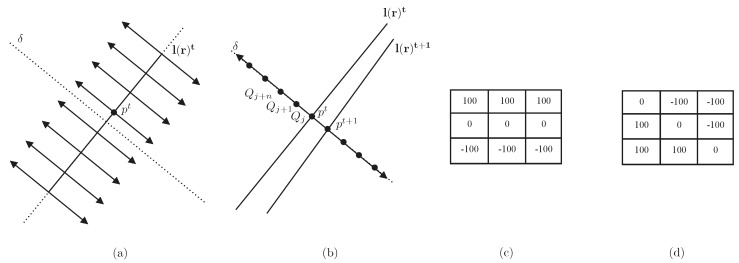
Moving edges (ME) algorithm to find correspondent points in image sequences. (**a**) Calculation of the direction *δ* normal to the line **l**(**r**)^*t*^; (**b**) search sampling {*Q_j_*, *j* ∈ [−*J*, *J*]} along the normal direction; (**c**) *M_δ_* mask at 180° and (**d**) *M_δ_* mask at 45°.

The algorithm consists of searching the correspondent point $p^{t+1}$ on line $l{\left(r\right)}^{t+1}$ in image $I^{t+1}$ of point $p^{t}$ in line $l{\left(r\right)}^{t}$. The search for a match is performed in the direction normal to the line $l{\left(r\right)}^{t}$, given by *δ*. For each point $p^{t}$, a search interval $\left\{Q_{j},j\in \left[-J,J\right]\right\}$ is defined. Each sample $Q_{j}$ is evaluated by the criterion $\zeta_{j}$. This evaluation consists in computing the convolution value between an image patch at the neighborhood *ν* of $Q_{j}$, and the mask $M_{\delta }$, which is a function of the orientation of line $l{\left(r\right)}^{t}$. The algorithm is shown in Figure 9.

Thus, the position of point $p^{t+1}$ on line $l{\left(r\right)}^{t+1}$ in image $I^{t+1}$ is given by:

(19) $$Q_{j^{*}}=\arg\max_{j\in \left[-J,J\right]}\zeta_{j}with=\left|I_{\nu \left(p^{t}\right)}^{t}*M_{\delta }+I_{\nu \left(Q_{j}\right)}^{t+1}*M_{\delta }\right|$$

A list of *k* points is produced, from which the segment extremities $s=\left(u_{1},u_{2}\right)$ are extracted.

One way to express the measurement **z** of the matched segment with respect to the expected prediction of it is to compute the orthogonal distance of the matched end points $u_{1}$ and $u_{2}$ to the predicted line **l**, as shown in Equation (16) and Figure 4.

By defining line measurement in this way, the matching can be accomplished regardless of which points of the corresponding line were detected by the tracker and of the segment length.

## 4. Experiments

This section includes the experimental part that tests the three main contributions of this article. The first part deals with the back-end and consists of a comparative evaluation between different landmark parametrizations. A set of simulations tests the benefits of the combination of point and line landmarks in the same map. The following part deals with the implementation of the developed segment-based SLAM front-end that includes the line segment active-search process presented in this paper. Finally, a complete heterogeneous landmark-based SLAM experiment that integrates the contributions to back-end and front-end is included.

### 4.1. Simulation of the Back-End for Heterogeneous SLAM

The point and line parametrizations previously presented have been tested independently in previous studies, such as [25,29,30]. This section offers a comparison of different heterogeneous approaches, including combinations of distinct landmarks on the same map. The purpose is to show the benefits of working with a heterogeneous parametrization that combines points and lines in a single map. The combinations performed are enumerated below:

1. Anchored homogeneous point (AHP)
2. Plücker line (PL)
3. Anchored homogeneous-points line (AHPL)
4. AHP + PL
5. AHP + AHPL

The MATLAB® EKF-SLAM toolbox [44] was extended with the heterogeneous functionality to perform the simulations.

**Figure 10:**
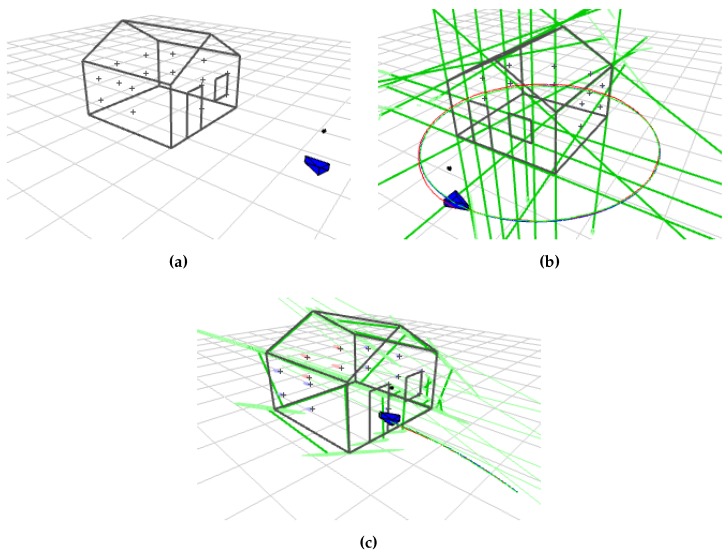
Environment world used for the simulation experiments of heterogeneous point and line SLAM. (**a**) Initial state of the environment world; (**b**) environment after performing circular trajectory with anchored homogeneous point (AHP) + Plücker line (PL) parametrization; (**c**) environment after performing approaching trajectory with AHP + AHPL parametrization.

Figure 10a shows the simulation environment. It consists of a house conformed by 23 lines and an array of 16 points distributed uniformly among the walls.

The robot performs two different trajectories. The first one is a circular path of 5 m in diameter, with a pose step of 8 cm and 0.09°. The second is a motion of 70 steps of 4 cm, each taken towards the scene. The linear noise is 0.5 cm and the angular noise 0.05°.

Besides the heterogeneous landmark capability of the toolbox, the transparency of the objects in the scene was also considered. By default, objects in the simulation environment of the toolbox are transparent, so landmarks are visible on almost every image frame. To work in a more realistic manner, an aspect graph was implemented to only observe visible surfaces of the house at each camera pose.

**Figure 11:**
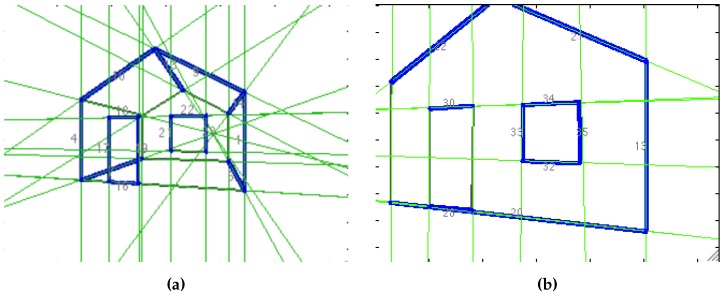
Different visualization modes of objects in the simulation environment. (**a**) Sensor view considering transparent objects; (**b**) sensor view considering opaque objects.

Both transparent and opaque object visualizations are shown in Figure 11. An example of a heterogeneous map constructed after a complete turn of the robot around the house is shown in Figure 10b. The parametrization used is AHP + PL. For the case of the approaching trajectory, a final heterogeneous map constructed is shown in Figure 10c. The parametrization used is AHP + AHPL. They display in green the line landmarks estimated and in blue the point landmarks. Real, predicted and estimated robot trajectories are displayed in blue, red and green, respectively.

For the circular path, a trajectory of five turns, considering transparent and opaque objects, was performed for each parametrization.

### 4.2. Integration of Line Segment Active-Search to the SLAM Front-End

The line-based SLAM front-end that was developed and that implements the line segment active-search presented in this article is covered in this section. The LSD and ME algorithms were applied to an image sequence showing a piece of furniture inside a room.

**Figure 12:**
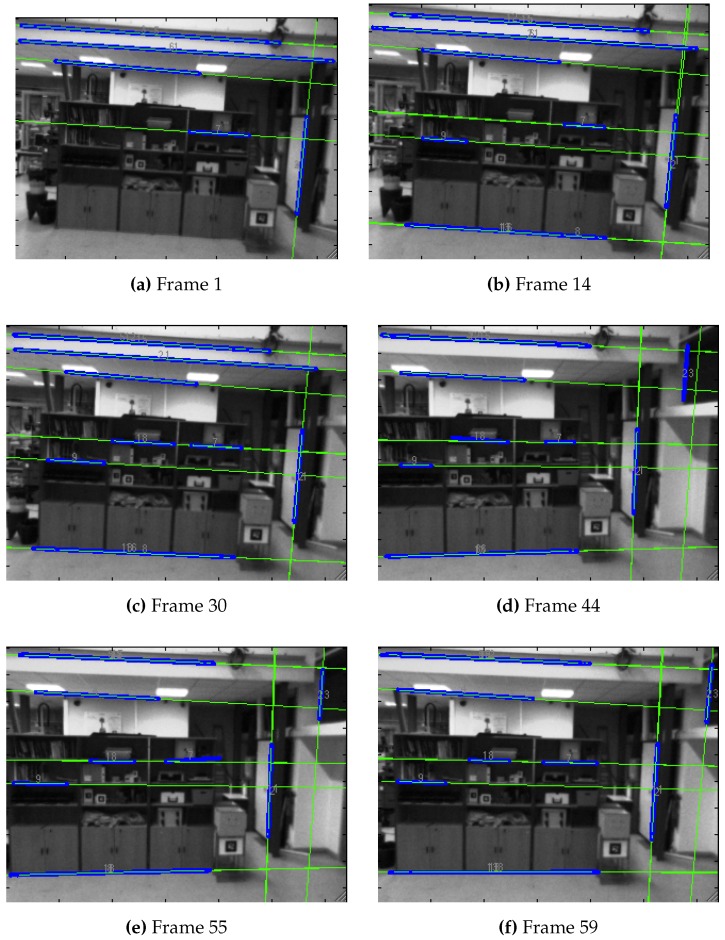
Front end operation of segment-based EKF-SLAM in an indoor sequence, showing the prediction and observation of landmarks. Infinite thin lines represent the estimated position of the landmark in the current image, while thicker segments show the match found.

Figure 12 shows the operation of this segment-based front-end of an EKF-SLAM process. Infinite thin lines represent the estimated position of the landmark in the current image, while thicker segments show the match found. It can be observed that the match corresponds to the estimation in most of the cases.

### 4.3. Heterogeneous SLAM Experiment

The complete heterogeneous SLAM solution was tested with the experiment described in this section.

The mobile robot used was a CRS Robotics F3 system, with a Microsoft LifeCam Studio camera mounted on it, along with a 9DOF Razor IMU that provided the robot state estimation at each frame. A total of 401 images with a 1280 × 720 resolution form the sequence. From this robot, it was possible to get the ground truth information with a repeatability of ±0.05 mm. The robot described a total trajectory of 0.4653 m. To get a prediction of the motion, the information provided by the IMU was used in a constant acceleration motion model. The inclusion of this additional sensor made it possible to cope with the inherent scale ambiguity of monocular systems.

**Figure 13:**
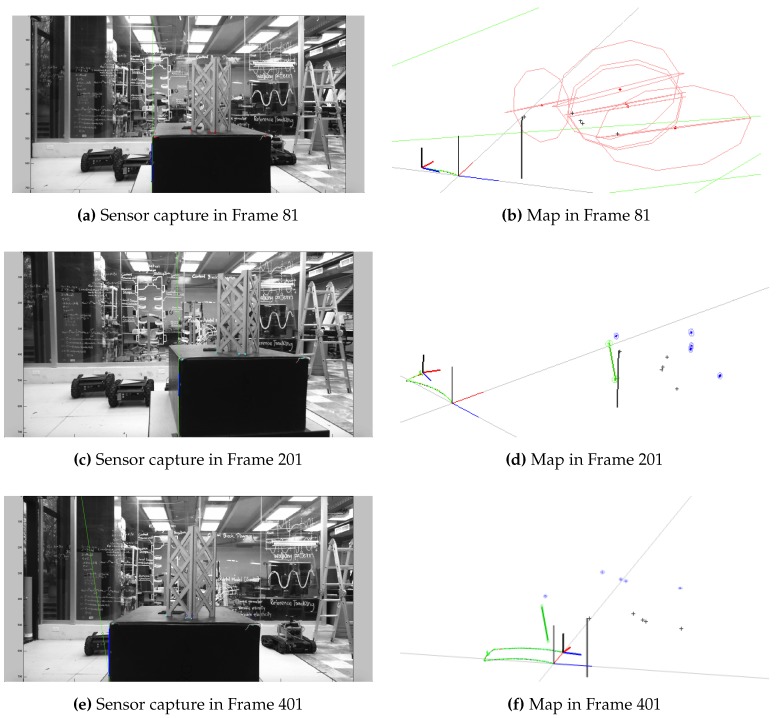
Point and line heterogeneous EKF-SLAM frames. (**a**,**c**,**e**) The front-end part; infinite thin lines represent the estimated position of the landmark in the current image, while thicker segments show the match found; circles represent the prediction of points, and points themselves are the match found; (**b**,**d**,**f**) The maps created on each frame, with the landmarks’ estimation, the current pose of the camera represented by the small reference frame and the trajectory described.

Figure 13 presents certain frames of the sequence, showing the landmarks used to update the state of the map. The AHP and AHPL were the parametrizations selected for the experiments, as they were the ones that provided better simulation results.

## 5. Results and Discussion

For the analysis, root mean square error (RMSE) evaluation is used to compute errors. At each instant *k*, the estimated position $\left(x_{k},y_{k},z_{k}\right)$ is compared to the ground truth position $\left({\hat{x}}_{k},{\hat{y}}_{k},{\hat{z}}_{k}\right)$.

(20) $$\epsilon_{k}=\sqrt{\left(\left({\left(x_{k}-{\hat{x}}_{k}\right)}^{2}+{\left(y_{k}-\hat{y_{k}}\right)}^{2}+{\left(z_{k}-{\hat{z}}_{k}\right)}^{2}\right)\right)}$$

From the previous results, the mean and standard deviation of the error are computed as follows:

(21) $$\begin{array}{c} \mu_{\epsilon }=\frac{1}{N}\sum_{k=1}^{N}\epsilon_{k} \end{array}$$

(22) $$\begin{array}{c} \sigma_{\epsilon }=\sqrt{\frac{1}{N}\sum_{k=1}^{N}{\left(\epsilon_{k}-\mu_{\epsilon }\right)}^{2}} \end{array}$$

Simulation of the back-end for heterogeneous SLAM was intended to compare the different parametrizations and to show the benefits of landmark heterogeneity.

The parametrization with the highest error is the Plücker line. Anchored parametrizations achieved the best performance, for both points and lines. There is an improvement effect in line parametrizations by the addition of points. Even for the anchored cases, already having a relative good performance while working independently, the heterogeneity improves the results, in such a way that the combination of both AHP and AHPL is the one with the least error along the simulated trajectories.

**Figure 14:**
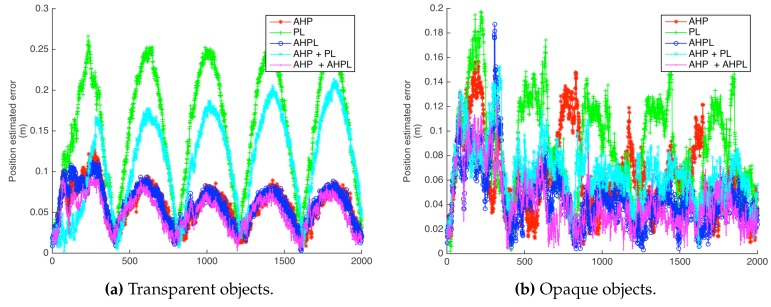
Robot position estimation errors for the circular trajectory.

**Figure 15:**
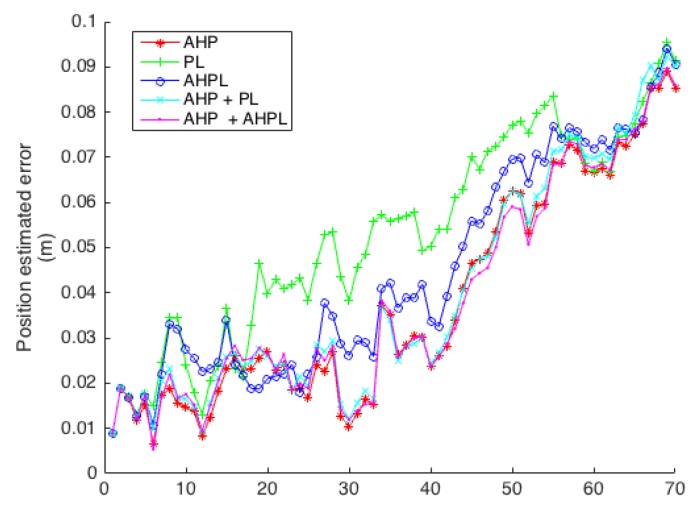
Robot position estimation errors for the approach trajectory.

The position error of the robot in the case of a circular trajectory with transparent and opaque objects is shown in Figure 14; these results are summarized in Table 1. For the case of the approaching trajectory, the results are shown and summarized in Figure 15 and Table 2.

**Figure 16:**
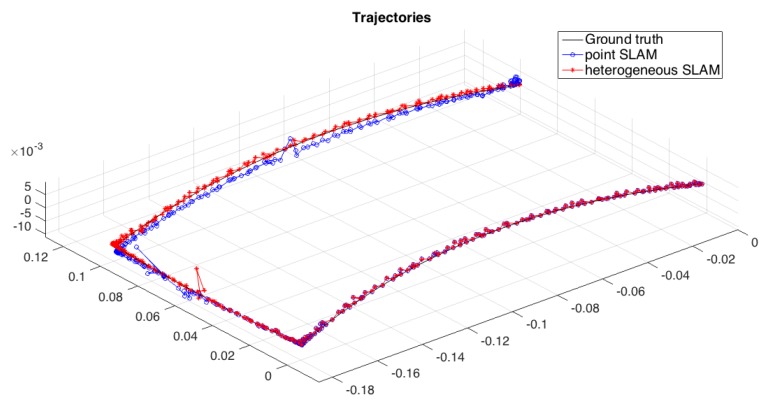
Ground truth, point SLAM estimated and heterogeneous SLAM estimated trajectories.

The complete SLAM experiment integrating the back-end with the developed front-end is used to compare the heterogeneous approach with a classic point-based SLAM applied to the same sequence. The ground truth and estimated trajectories for each SLAM approach tested are shown in Figure 16.

**Figure 17:**
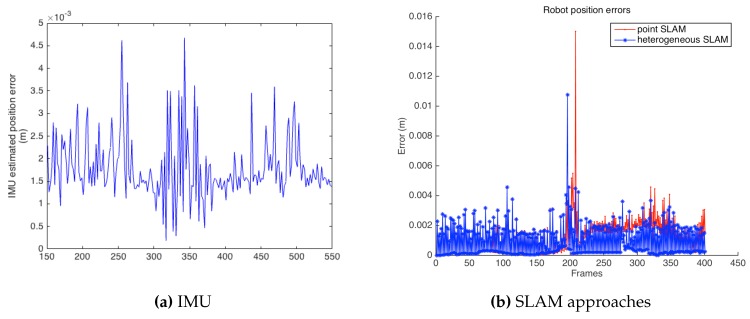
Robot position estimation error comparison between IMU, point SLAM and heterogeneous SLAM approaches.

Figure 17 presents results in terms of the robot position estimation error, comparing the IMU estimation to both point and heterogeneous SLAM. As can be observed, the heterogeneous approach results in lower errors, as previously presented in the simulation part. Near Frame 200, there was a change in the motion direction of the robot, which can be seen as a peak in the position estimation error graph. Even in this case, heterogeneous SLAM achieved a better performance than the point-based SLAM. Table 3 shows a comparative summary of the errors from the three cases.

## 6. Conclusions

The purpose of this paper is to prove the benefits of including heterogeneous landmarks when building a map from an EKF-based visual SLAM method. Several authors have shown interest in the use of heterogeneous landmarks. For the front-end, the interest for joint tracking of points and lines is found in [45], while for the back-end, a theoretical study is presented in [31], and preliminary results of an EKF-based SLAM method, based on heterogeneous landmarks, are presented in [46]. The experiments performed that the authors describe have shown that the robot localization or the SLAM stability can be improved by combining several landmarks, *i.e.*, points and lines. The use of just monocular vision provides only partial observations of landmarks by features extracted from images; here, undelayed initialization of landmarks is used, as was proposed initially by Solà *et al.* [25,29] for points and lines. The use of simulated data has shown how the choice of the landmark representation has an impact on the accuracy of the map. The best ones, considering the construction of a map with heterogeneous landmarks, are anchored homogeneous points and anchored homogeneous points lines. These parametrizations were used in a complete heterogeneous SLAM experiment that produced better results than the classic point-based case, by reducing the camera position estimation error.

Another contribution of this paper is a method proposed for a segment-based SLAM front-end. This method relies on the line segment active-search process presented in this article and on state-of-the-art line detection and matching processes. The methods that compose the developed front-end that resulted were discussed, recalling, first, their theoretical background and, then, presenting some experimental evaluations on image sequences that show the stability of the process.

Finally, a complete heterogeneous landmark-based SLAM experiment was presented, integrating the contributions with the back-end and the front-end and confirming the results obtained independently.

In future work, constraints will be exploited in the map, typically when points and lines are extracted from known portions of the scene.

## Figures and Tables

**Table 1:** Robot position error mean and standard deviation, for transparent and opaque object visualization, for the different heterogeneous landmark parametrizations in the circular trajectory in the simulated environment.

		AHP	PL	AHPL	AHP + PL	AHPL + AHPL
Mean	Transparent objects	0.05856	0.1532	0.05861	0.1109	0.04982
	Opaque objects	0.06332	0.08806	0.04561	0.06634	0.04114
Standard Deviation	Transparent objects	0.02426	0.07139	0.02268	0.05733	0.02025
	Opaque objects	0.0316	0.03961	0.0236	0.02298	0.02368

**Table 2:** Robot position error mean and standard deviation for the different heterogeneous landmark parametrizations in the approach trajectory in the simulated environment.

	AHP	PL	AHPL	AHP + PL	AHP + AHPL
Mean	0.038	0.053	0.044	0.040	0.038
Standard Deviation	0.024	0.023	0.024	0.025	0.023

**Table 3:** Robot position error mean, maximum, minimum and standard deviation, for IMU estimation, point and heterogeneous SLAM solutions in the real environment.

	Mean	Max	Min	SD
IMU	0.0018	0.0047	0.0002	0.0007
Point SLAM	0.0011	0.1500	0.0000	0.0012
Heterogeneous SLAM	0.0008	0.0107	0.0000	0.0011
